# Identification of FMRP target mRNAs in the developmental brain: FMRP might coordinate Ras/MAPK, Wnt/β-catenin, and mTOR signaling during corticogenesis

**DOI:** 10.1186/s13041-020-00706-1

**Published:** 2020-12-16

**Authors:** Cristine R. Casingal, Takako Kikkawa, Hitoshi Inada, Yukio Sasaki, Noriko Osumi

**Affiliations:** 1 Department of Developmental Neuroscience, United Center for Advanced Research and Translational Medicine (ART), Tohoku University Graduate School of Medicine, 2-1 Seiryo-machi, Aoba-ku, Sendai, Miyagi 980-8575 Japan; 2 Laboratory of Health and Sports Sciences, Division of Biomedical Engineering for Health and Welfare, Tohoku University Graduate School of Biomedical Engineering, 6-6-12, Aramaki Aza Aoba Aoba-ku, Sendai, Miyagi 980-8579 Japan; 3 Functional Structure Biology Laboratory, Department of Medical Life Science, Yokohama City University Graduate School of Medical Life Science, 1-7-29 Suehiro-cho, Tsumuri-ku, Yokohama, 230-0045 Japan

**Keywords:** RNA binding protein, FMRP, FXS, mRNA targets, Corticogenesis

## Abstract

Corticogenesis is one of the most critical and complicated processes during embryonic brain development. Any slight impairment in corticogenesis could cause neurodevelopmental disorders such as Fragile X syndrome (FXS), of which symptoms contain intellectual disability (ID) and autism spectrum disorder (ASD). Fragile X mental retardation protein (FMRP), an RNA-binding protein responsible for FXS, shows strong expression in neural stem/precursor cells (NPCs) during corticogenesis, although its function during brain development remains largely unknown. In this study, we attempted to identify the FMRP target mRNAs in the cortical primordium using RNA immunoprecipitation sequencing analysis in the mouse embryonic brain. We identified 865 candidate genes as targets of FMRP involving 126 and 118 genes overlapped with ID and ASD-associated genes, respectively. These overlapped genes were enriched with those related to chromatin/chromosome organization and histone modifications, suggesting the involvement of FMRP in epigenetic regulation. We further identified a common set of 17 FMRP “core” target genes involved in neurogenesis/FXS/ID/ASD, containing factors associated with Ras/mitogen-activated protein kinase, Wnt/β-catenin, and mammalian target of rapamycin (mTOR) pathways. We indeed showed overactivation of mTOR signaling via an increase in mTOR phosphorylation in the *Fmr1* knockout (*Fmr1* KO) neocortex. Our results provide further insight into the critical roles of FMRP in the developing brain, where dysfunction of FMRP may influence the regulation of its mRNA targets affecting signaling pathways and epigenetic modifications.

## Introduction

The neocortex is an important region in higher cognitive functions, and its formation, i.e., corticogenesis, is an extremely complicated process during embryonic brain development. During corticogenesis, neural stem/precursor cells (NPCs), or in another name, radial glial cells (RGCs) proliferate and differentiate to immature neurons. These immature neurons migrate towards the basal side and stack from inside-to-outside, and further differentiate into glutaminergic excitatory neurons, produce neurotransmitters or neurotrophic factors, and begin to form neural networks [1, 2]. These processes are precisely programmed at the genetic level; therefore, any slight impairment in the developmental program could result in severe functional defects in the brain.

Intensive genetic analyses of patients with neurodevelopmental disorders have identified various molecules critical for the neuropathogenesis [3, 4]. *Fragile* *X* *mental* *retardation* *1* (*FMR1*) encoding fragile X mental retardation protein (FMRP) is a well-characterized gene related to a typical neurodevelopmental disorder, Fragile X syndrome (FXS) [5, 6]. FXS patients have intellectual disability (ID), and  25% of male and 6% of female FXS patients show features of autism spectrum disorder (ASD) [7–9]. Therefore, elucidating the FMRP function is critical to understand the molecular mechanism relating also to ID and ASD.

FMRP is a polyribosome-associated RNA binding protein (RBP) [10, 11]. In the matured neuron of the adult brain, FMRP is localized at cell body, proximal dendrites, and axons [12, 13]. FMRP plays profound regulatory roles in the synaptic function and neuronal plasticity through the interaction with transcripts of pre- and postsynaptic proteins [14, 15] and by regulation of mRNA trafficking into the dendrite [11, 16]. On the other hand, FMRP is also expressed in the RGCs and immature neurons of the developing brain [17, 18]. Within the RGCs, FMRP is localized at the apical and basal endfeet [17, 18]. Previous studies have suggested that FMRP regulates the transition from RGCs to intermediate progenitors in the embryonic brain [18] and that its deficit affects neuronal migration and cortical circuitry [19]. Altogether, FMRP has multiple roles at distinct time points in brain development.

Since the discovery of FMRP, various studies have been conducted to identify FMRP target genes using RNA-binding protein immunoprecipitation (RIP) [17, 20], crosslinking immunoprecipitation [10], photoactivatable ribonucleoside-enhanced crosslinking and immunoprecipitation [21] and ribosome profiling [22], all of which are based on high-throughput sequencing. While the role of FMRP and its target genes have been most highlighted in the adult brain [10, 20, 23, 24], only limited studies have reported its role in the embryonic brain [17, 18]. In this study, we performed RIP high-throughput sequencing (RIP-seq) analysis using mouse embryonic brain samples and identified FMRP target genes that are also associated with ID and ASD. We also found FMRP “core” target genes shared with our data, neurogenesis, ID, and ASD, which were involved in the Ras/mitogen-activated protein kinase (MAPK), Wnt/β-catenin, and mTOR pathways. Our data may contribute to understand the role of FMRP in corticogenesis and may serve as important resources for future studies of neurodevelopmental disorder.

## Results

### FMRP is expressed in the mouse embryonic cortex

**Fig. 1:**
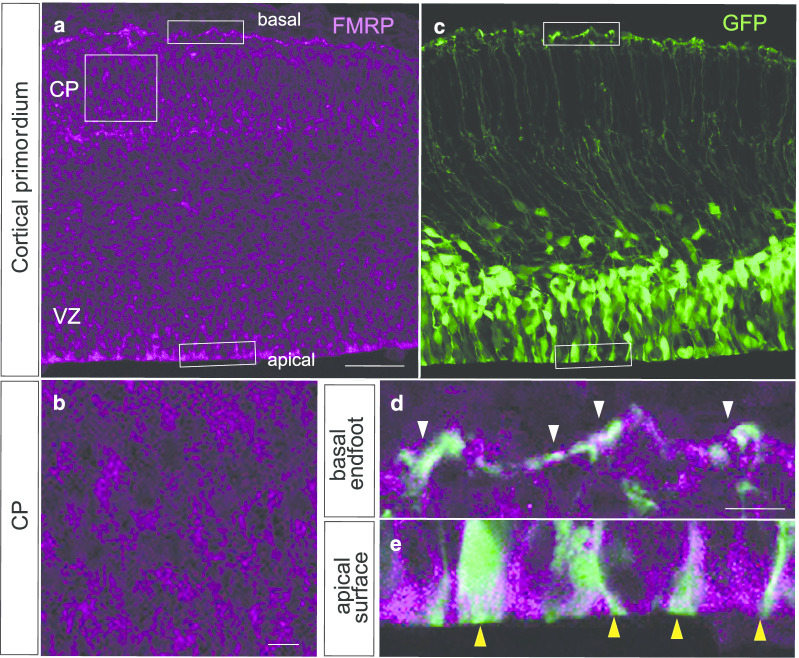
FMRP expression pattern in the coronal sections of the E14.5 WT mouse neocortex. **a**, **b** FMRP is expressed throughout the cortical primordium, showing the highest accumulation at both basal and apical endfeet of the RGCs. **c** Green fluorescent signal shows GFP-labeled RGCs. **d**, **e** Merged images showed that FMRP is highly localized in the basal (white arrowheads) and apical (yellow arrowheads) endfeet of the RGCs. *CP* cortical plate, *VZ* ventricular zone. Scale bars: 50 μm, 10 μm (inset)

We first confirmed the FMRP expression in the cortical primordium of wild type (WT) mice at embryonic day (E) 14.5 when massive neurogenesis occurs. The immunostaining signal of FMRP was present in the cortical plate (CP), including immature neurons, and at apical (ventricular) and basal (pial) surface areas (Fig. 1a, b). The accumulation of FMRP at the apical and basal endfeet of the RGCs was confirmed by the GFP-labeling of RGCs using in utero electroporation with an EGFP reporter gene (pCAG-EGFP) (Fig. 1c); FMRP was overlapped with GFP fluorescence in both the apical and basal endfeet of the RGCs (Fig. 1d,e). Therefore, our data are consistent with findings in the previous literature [17–19].

### Identification of FMRP target mRNAs in embryonic mouse cortex

**Fig. 2:**
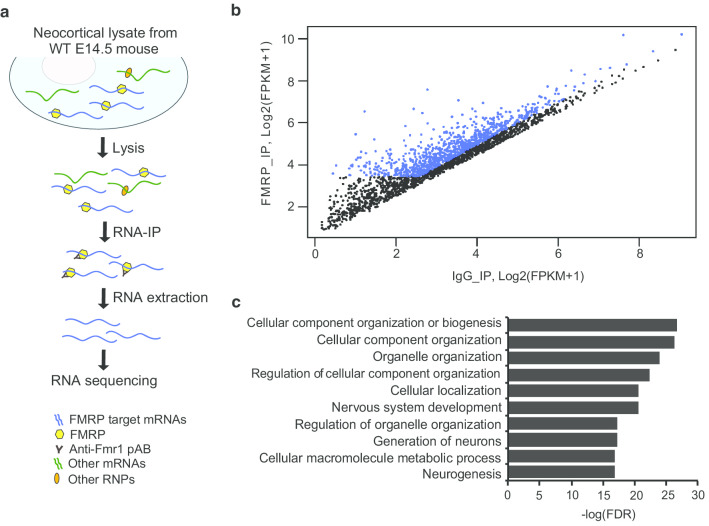
FMRP mRNA targets identified from the RIP-seq of the WT E14.5 cortex. **a** RIP-seq workflow. **b** Scatterplot comparing log2 ratios of significant (q < 0.01) FPKM expressions values of FMRP-IP and IgG-IP. The vertical coordinates represent the log2-FPKM + 1 values for each gene from FMRP-IP, and the horizontal coordinates represent the log2-FPKM + 1 values for each gene IgG-IP. Highlighted in blue are the 865 FMRP target genes selected based on gene expression values, log_2_FC > 1, and FPKM > 10 for the FMRP-IP. **c** The top 10 GO (biological process) terms enriched in the 865 FMRP target genes

To explore the target mRNAs of FMRP during corticogenesis, we performed RIP-seq analyses using cortical samples isolated from the WT mice at E14.5 (Fig. 2a). In total, we found 2288 candidate FMRP target mRNAs that were significantly expressed (measured by fragment per kilobase of transcript per RNA-seq read mapped, FPKM) in the FMRP-IP compared to IgG-IP or the negative control (FMRP-IP FPKM > IgG-IP FPKM). Next, we selected a stringent set of 947 mRNAs from 865 FMRP target genes based on gene expression values, fold changes in the logarithmic scale with base 2 greater than 1 (log_2_FC > 1), and FPKM greater than 10 against the FMRP-IP. (Fig. 2b; Additional file 1: Table S1). The set of 865 FMRP target genes showed higher gene expression than the negative control, and therefore, could be validated as targets of FMRP in the developing neocortex.

To estimate the functions of the FMRP target genes, we performed gene ontology (GO) analyses using the visual annotation display (VLAD)—gene analysis and visualization analysis tool of the Mouse Genome Informatics (MGI) [25]. The top significant GO terms included biological processes related to early brain development, such as “nervous system development,” “generation of neurons,” and “neurogenesis.” (Fig. 2c). This is quite reasonable because the identified FMRP target genes were collected from developing cortices where massive neurogenesis is occurring (Fig. 1, Additional file 2: Table S2).

Next, we compared our FMRP target genes in the embryonic mouse brain with those detected in the mouse cortex and cerebellum at postnatal (P) day 11–13 [10], in the hippocampus at P28 to 32 [26], and in the cultured adult NPCs derived from the dentate gyrus at 8 to 10-week [22]. We found hundreds of the genes were overlapped and significantly enriched (Additional file 3: Table S3), suggesting common functions of FMRP shared in the embryonic, postnatal and adult brains.

### Overlap among FMRP targets, neurogenesis, ID and ASD-associated genes

**Fig. 3:**
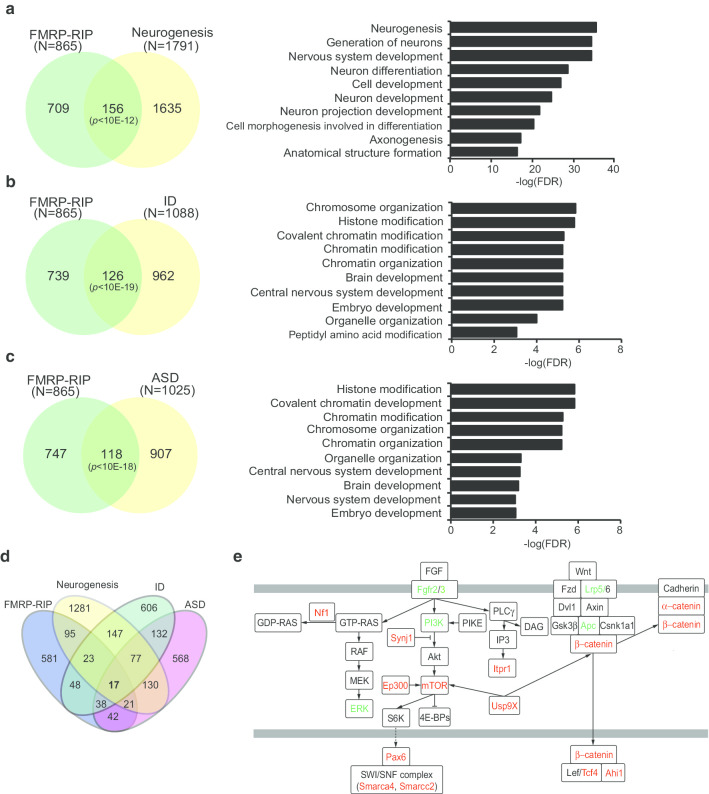
The overlap between our FMRP target genes and genes associated with neurogenesis, ID, and ASD and their involvement in signaling pathways. **a** Venn diagram depicting the overlap of our FMRP target genes and neurogenesis genes retrieved from the MGI (GO: neurogenesis) and its top 10 GO. **b** Venn diagram depicting the overlap of our FMRP target genes and ID-associated genes retrieved from the OMIM (keyword: ID) and its top 10 GO. **c** Venn diagram depicting the overlap of our FMRP target genes and ASD-associated genes retrieved from the SFARI database. Statistical significance was determined by hypergeometric distribution analysis. **d** The 17 FMRP “core” target genes common in the four gene sets FMRP-IP, neurogenesis, ID, and ASD. **e** Signaling pathways in which the FMRP target genes were involved. The highlighted molecules were the identified FMRP “core” target genes (red) and other FMRP target genes in our data (green), which were involved in Ras/MAPK, Wnt/β-catenin, and mTOR signaling pathways

To obtain more insight for the significance of FMRP target candidate genes, we focused on the three criteria, i.e., neurogenesis, ID, and ASD since FXS patients often show ID and ASD symptoms [8, 9]. We first compared the identified 865 FMRP target genes with 1791 neurogenesis genes from MGI [25]. There was a highly significant overlap of 156 genes between the two groups, including those mainly assigned to GOs related to “Neuronal development”, “Generation of neurons,” “Neuron differentiation”, and “Cell morphogenesis involved in differentiation” (Fig. 3a). The results thus indicate that several targets of FMRP are important for neurogenesis during early brain development. We also found genes for “Axonogenesis” and “Neuron projection development”, i.e., the events after neuronal differentiation, as GOs for FMRP target genes, which may suggest the importance of FMRP in the establishment of neuronal networks.

We then examined the association between the identified FMRP target genes with 1088 ID genes based on Online Mendelian Inheritance in Man (OMIM) [27], which resulted in 126 genes (Fig. 3b), as expected, because ID is a core feature of FXS [28]. These overlapped genes included not only GOs such as “Brain development” and “Central nervous system development”, but also “Chromosome organization” and “Histone modification” unexpectedly. We also found 118 FMRP target genes that significantly overlapped with the 1025 ASD-associated genes using the public database Simons Foundation Autism Research Initiative (SFARI) [29] (Fig. 3c); again there came up with GOs such as “Histone modification” and “Chromatin organization”, as well as “Brain development”. As we expected, these GOs include several syndromic ASD-associated genes such as *paired* *box* *6* (*PAX6*) [30, 31], *lysine* *acetyltransferase* *6a* (*KAT6A*) [32]*,* *mammalian* *target* *of* *rapamycin* (*mTOR*) [33]*,* *Abelson’s* *helper* *integration* *1* (*AHI1*) [34] and *ubiquitin-specific* *peptidase* *9* *X-linked* (*USP9X*) [35]. Overall, the overlap between FMRP target genes linked to ID and ASD could provide a correlation between loss of function of FMRP and the development of both ID and ASD.

Finally, we identified 17 genes as the FMRP “core” target genes shared with neurogenesis, ID, and ASD gene sets (Fig. 3d, Table 1). These FMRP “core” target genes contained not only major transcription regulators such as Pax6, Myt1l, and Tcf4 but also components of Ras/MAPK (Nf1) [36], Wnt/β-catenin (Ahi1, Ctnna2, and Ctnnb1) [34, 37], and mTOR (mTOR, Ep300, Itpr1 and Synj1) [38–41] signaling pathways (Fig. 3e). As mentioned above, the FMRP “core” target genes also included factors of the chromatin-remodeling complex [42], such as Nipbl, Smarcc2, and Smarca4. Besides, Usp9X has been thought to be involved in developmental processes through Wnt/β-catenin and mTOR pathways [43]. These common pathways can cause shared symptoms among FXS, ID, and ASD.

**Table 1.** 17 FMRP target genes associated with neurogenesis, ID and ASD

Gene symbol	Gene name
*Ahi1*	*Abelson* *Helper* *Integration* *Site* *1*
*Ank3*	*Ankyrin* *3*
*Ctnna2*	*Catenin* *Alpha* *2*
*Ctnnb1*	*Catenin* *Beta* *1*
*Ep300*	*E1A* *Binding* *Protein* *P300*
*Itpr1*	*Inositol* *1,4,5-Trisphosphate* *Receptor* *Type* *1*
*Mtor*	*Mechanistic* *Target* *Of* *Rapamycin* *Kinase*
*Myt1l*	*Myelin* *Transcription* *Factor* *1* *Like*
*Nf1*	*Neurofibromin* *1*
*Nipbl*	*NIPBL* *Cohesin* *Loading* *Factor*
*Pax6*	*Paired* *Box* *6*
*Smarca4*	*SWI/SNF* *Related,* *Matrix* *Associated,* *Actin* *Dependent* *Regulator* *Of* *Chromatin,* *Subfamily* *A,* *Member* *4*
*Smarcc2*	*SWI/SNF* *Related,* *Matrix* *Associated,* *Actin* *Dependent* *Regulator* *Of* *Chromatin* *Subfamily* *C* *Member* *2*
*Synj1*	*Synaptojanin* *1*
*Tbc1d23*	*TBC1* *Domain* *Family* *Member* *23*
*Tcf4*	*Transcription* *Factor* *4*
*Usp9x*	*Ubiquitin* *Specific* *Peptidase* *9* *X-Linked*

### Expression of the FMRP “core” target genes in the developing cortex

**Fig. 4:**
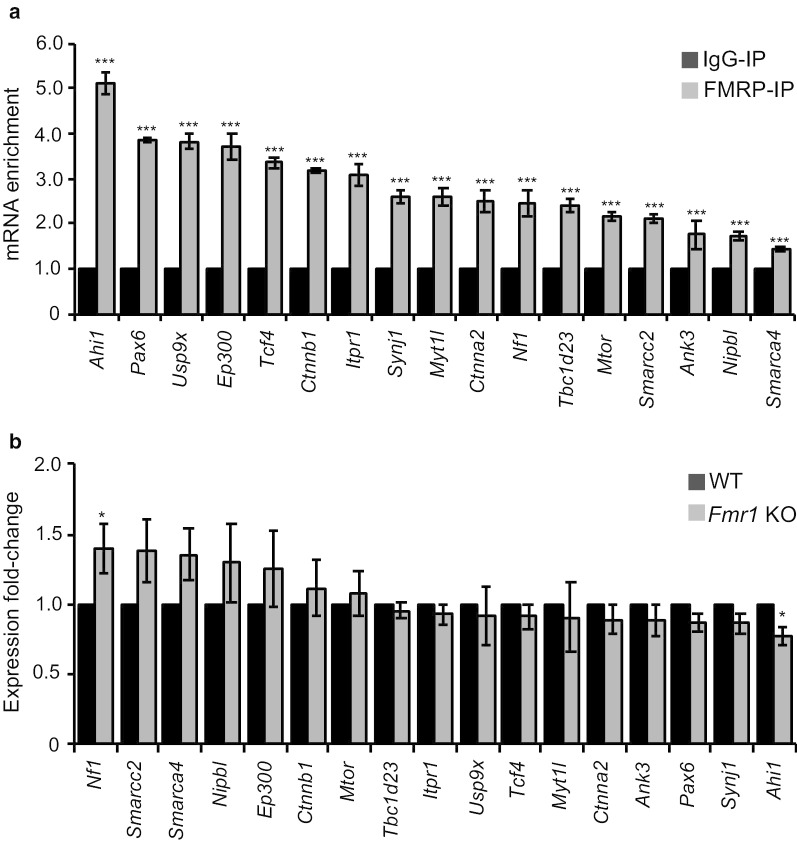
Validation of the 17 FMRP “core” target genes by RIP-qPCR and their expression in the WT and *Fmr1* KO mice. **a** The RNAs from WT (n = 3) of the E15.5 mouse dorsal telencephalons were isolated and subjected to cDNA synthesis and RT-qPCR. All 17 FMRP “core” target genes were significantly enriched in the FMRP-IP compared with that of IgG-IP (n = 3). **b** The RNAs (n = 7 WT; n = 7 *Fmr1* KO) from the E15.5 mouse dorsal telencephalon were isolated and subjected to cDNA synthesis and RT-qPCR. *Nf1* and *Ahi1* showed an increase and decrease in expression, respectively, in *Fmr1* KO neocortex, compared to the WT. The Student’s *t-*test was used to test statistical significance (*p < 0.05, ***p < 0.0001). Error bars represent the standard error of the mean (SEM)

To confirm the FMRP interaction with the mRNAs of the 17 FMRP “core” target genes, we performed RIP-qPCR. All mRNAs were significantly enriched in the FMRP-IP, suggesting that these mRNAs are targeted by FMRP (Fig. 4a). We further examined the mRNA amount of the genes in cortical primordial samples from E15.5 WT and *Fmr1* knockout (KO) male mice. We found a significant increase of *Nf1* mRNA and a significant decrease of *Ahi1* mRNA in the *Fmr1* KO mouse neocortex, while other genes showed no significant difference (Fig. 4b). These findings suggest that FMRP mainly functions as a post-transcriptional regulator of its target genes.

**Fig. 5:**
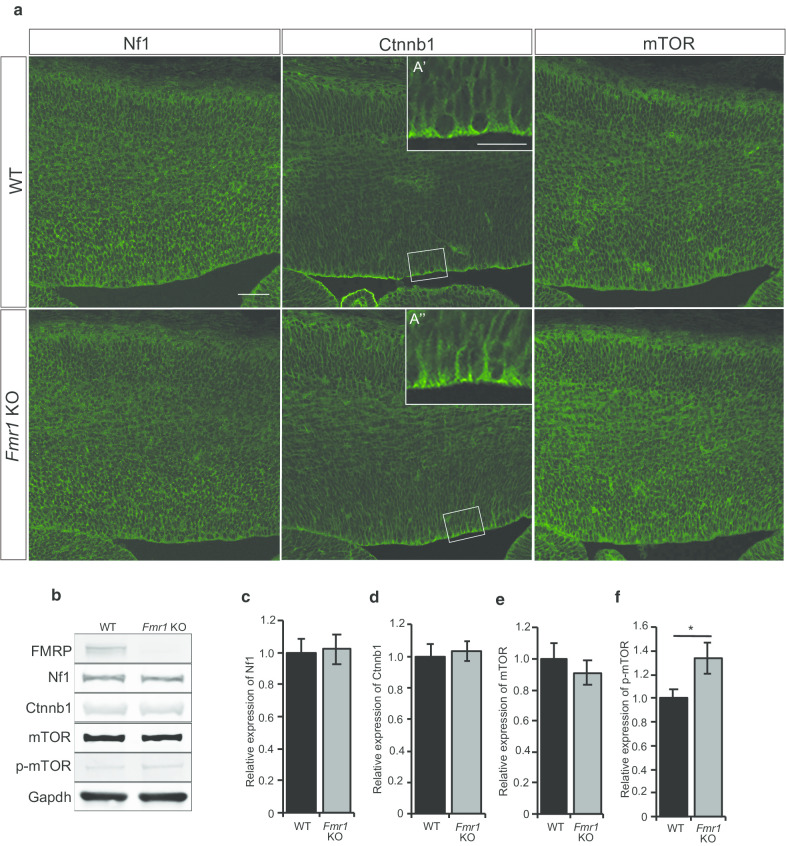
Nf1, Ctnnb1 and mTOR expression in mouse neocortical lysates. **a** Expression pattern of Nf1 (n = 5 WT; n = 5 *Fmr1* KO), Ctnnb1 (n = 6 WT; n = 6 *Fmr1* KO) and mTOR (n = 5 WT; n = 5 *Fmr1* KO) in E15.5 neocortex showed no difference between the WT and *Fmr1* KO mice. **b** Representative western blots of selected FMRP target genes. **c**–**f** Relative expression of Nf1 (n = 12 WT; n = 12 *Fmr1* KO), Ctnnb1 (n = 7 WT; n = 7 *Fmr1* KO), mTOR (n = 12 WT; n = 12 *Fmr1* KO) and p-mTOR (n = 10 WT; n = 10 *Fmr1* KO) were measured using ImageJ software. The findings indicate no difference in Nf1, Ctnnb1 and mTOR expression between WT and *Fmr1* KO neocortices. However, p-mTOR at Ser2448 showed a 25.4% increase in protein level in the *Fmr1* KO. Scale bars: 50 μm, 20 μm (inset). Student’s *t-*test was used to test statistical significance (*p < 0.05). Error bars represents the SEM

Among the 17 FMRP “core” target genes, we highlighted three genes, *Nf1,* *Ctnnb1*, and *Mtor*, because these are involved in Ras/MAPK, Wnt/β-catenin, and mTOR pathways, respectively [40, 44, 45]. We first assessed the protein expression of Nf1, Ctnnb1, and mTOR in the cortical primordium at E15.5 (Fig. 5a, b). The Nf1 and mTOR proteins were widely expressed throughout the cortical primordium, while Ctnnb1 was concentrated at the apical surface. There seemed to be no change in these expression patterns in *Fmr1* KO mice compared to that of WT (Fig. 5a). Immunoblotting analyses also showed that Nf1, Ctnnb1, and mTOR showed normal expression levels in *Fmr1* KO mice corresponding to the immunostaining (Fig. 5a–e). This could imply that the translation of these targets was unaffected in the *Fmr1* KO neocortex. Although the protein level of mTOR was unchanged in the *Fmr1* KO, its phosphorylated form (p-mTOR) at Ser2448 was significantly elevated by 25.4% in the lysate of the *Fmr1* KO neocortex compared to that of WT (Fig. 5b, f). This result suggests that mTOR signaling might be enhanced in *Fmr1* KO mice during corticogenesis, which is similar to the result in the adult hippocampus [40] but the first evidence in the embryonic brain.

## Discussion

Even though FMRP shows unique expression patterns in the cortical primordium, most of the previous studies have highlighted FMRP’s role in the post-transcriptional regulation of its mRNA targets in the postnatal and adult brain. Here we focused on the FMRP target genes during corticogenesis and identified 865 genes. Importantly, they overlapped with those identified in the postnatal and adult brains, suggesting that FMRP have common targets at different developmental stages. It is of note that the 865 FMRP target genes included not only those related to neural and neuronal development, as expected, but also those involved in chromatin remodeling and histone modifications. This emphasizes involvement of FMRP in epigenetic regulation in the developing and adult brain.

Using independent databases, i.e., OMIM and SFARI, we showed that our FMRP target genes significantly overlapped with genes associated with ID and ASD. This may suggest that phenotypes shared among FXS, ID, and ASD patients may likely be caused by these common genes. In other words, these lists also represent common impaired pathways or molecular mechanisms observed in neurodevelopmental disorders.

We identified 17 FMRP “core” target genes common in FXS, ID, ASD, and neurogenesis gene sets including critical components in Ras/MAPK, Wnt/β-catenin, and mTOR pathways. Nf1 is a negative regulator of the Ras/MAPK signaling pathway, and loss of Nf1 leads to an increase in the number of NPCs, but not of neurons, within the mouse cortex [36]. Several groups have previously shown Nf1 as a target of FMRP [10, 21, 22, 46] with some inconsistency. Knocking down *Fmr1* in oocytes increases *Nf1* at the mRNA level but significantly decreases its protein level [46]. Ribosomal profiling of cultured adult NPCs shows normal *Nf1* mRNA and protein expression in the *Fmr1* KO [22]. In our study using *Fmr1* KO forebrain samples, the absence of FMRP caused an increased expression of *Nf1* mRNA but unaffected protein amount. Thus, regulation of Nf1 expression at mRNA and protein levels may be highly context-dependent; the increase of *Nf1* mRNA level could be an indirect effect of FMRP on upstream transcriptional or epigenetic regulators of *Nf1* that might be different in distinct cell types*.*

It is well known that the Wnt/β-catenin pathway regulates NPC proliferation and neuronal differentiation in the developing mouse neocortex [47–49]. In this study, we identified a Wnt/β-catenin pathway regulator, *Ctnnb1*, as a target of FMRP. However, *Ctnnb1* mRNA and protein amounts were normal in *Fmr1* KO embryonic brain. Although Ctnnb1 amount is reported to be reduced in adult NPCs derived from *Fmr1* KO mouse [50], NPCs derived from human embryonic stem cells established from FXS patients showed unchanged Ctnnb1 level compared to the nonaffected control [51]. These discrepancies might come from different stages of the NPCs, and our finding is rather close to the NPCs that may reflect FXS conditions during development.

mTOR signaling is a well-known pathway for its responsibility to not only for FXS [40], but also for ASD [52, 53]. Overactivation of mTOR signaling during neurogenesis can increase protein synthesis and induce neuronal differentiation [54, 55], leading to cortical malformation [56]. Several studies have explored the relationship between FMRP and mTOR signaling in the postnatal and adult mouse brains; mTOR signaling is exaggerated in the absence of FMRP [40]. Here, we identified several components of the mTOR-related pathway as FMRP target genes and found for the first time that the p-mTOR level was elevated in the developing neocortex of *Fmr1* KO mice. Our finding thus suggests importance of FMRP-mTOR pathways shared in the embryonic and postnatal brains.

Our FMRP “core” target genes contain not only mTOR, but also two possible regulators of mTOR signaling, i.e., Synj1 and Ep300. Synj1 is an inositol phosphatase expressed in neuronal synaptic terminals, which is essential for the neuronal function, survival and differentiation [38]. Synj1 may affect activity of mTOR signaling by regulating a membrane phosphatidylinositol-4,5-bisphosphate, of which reduction could decrease in mTOR signaling activity [38]. While acetyltransferase Ep300 is a transcriptional co-activator significantly elevated in *Fmr1* KO hippocampal NPCs [57], it can positively regulate mTORC1 activity through acetylation of Raptor, a negative regulator of mTORC1 [41]. We did not confirm protein levels of Synj1 and Ep300 because there was no significant change in mRNA levels between the WT and *Fmr1* KO mice. However, gene expression of Synj1 and Ep300 exhibited slight decrease and increase, respectively, in *Fmr1* KO mice. Since Synj1 and Ep300 can work as negative and positive regulators of mTOR signaling, respectively, the slight changes in mRNAs of these genes might synergistically result in a synergistic effect on the enhanced p-mTOR level.

Another possible player for the increased p-mTOR level might be PIKE (also known as Centg1, centaurin gamma 1), an upstream activator of mTOR and a target of FMRP [40, 58]. Stimulation of the group I metabotropic glutamate receptors activates PI3K-mTOR activity through PIKE [59, 60]. In the hippocampal neurons of *Fmr1* KO mice, PIKE is elevated enhancing the activity of PI3K, which then increases phosphorylation of mTOR, leading to the overactivation mTOR signaling. Interestingly, mTOR signaling can enhance phosphorylation of mTOR downstream targets S6K and 4E-BPs [40]. S6K activity induces differentiation in pluripotent human embryonic stem cells [54], while knockdown of 4E-BP2, the major 4E-BP expressed in the brain, is sufficient to induce NPCs differentiation [61]. Taken together, our results can explain how altered proliferation and differentiation of NPCs [18, 62] may lead to an abnormal cortical cytoarchitecture seen in *Fmr1* KO mouse brain [63].

It is previously known that FMRP regulates translation of its target genes [reviewed in refs. 64–67]. For example, a subset of FMRP target genes including *Icam5* (*intercellular* *adhesion* *molecule* *5*) [68], *Gsk3β* (*glycogen* *synthase* *kinase* *3* *beta*) [50] and glutamate receptor subunits [69] have shown increased protein levels in *Fmr1* KO mice. In the present study, however, protein levels of Nf1, Ctnnb1, and mTOR were normal in the *Fmr1* KO neocortex, which is similar to previous results regarding other FMRP target genes such as *Psd-95* (also known as *Dlg4,* *discs* *large* *MAGUK* *scaffold* *protein* *4*) [70], *Snap25* (*synaptosome* *associated* *protein* *25*) [71] and *Cyfip1*(*cytoplasmic* *FMR1* *interacting* *protein*) [72]. This suggests that FMRP may not directly regulate translation of these target genes; small changes in expression of these target genes can be attributed to RNA stability [70] and/or transcriptional epigenetic regulators such as Brd4 (bromodomain containing 4) [73]. Thus, FMRP is a multifunctional protein that can regulate its target genes in a context-dependent manner.

Finally, FMRP may further affect gene expression during corticogenesis by regulating epigenetic (chromatin and histone) modifications (as seen in Fig. 3b, c, Additional file 2: Table S2). One of the chromatin targets of FMRP, bromodomain-containing 4, Brd4, has been reported to be overactivated in *Fmr1* KO mice, and its inhibition alleviated phenotypes in the mouse associated with FXS [73]. The mechanism of regulation by FMRP on these epigenetic regulators was not investigated in this study. Whether or not the misregulation of epigenetic modifications is due to the absence of FMRP, it is evident that these modifications could modulate widespread changes in the expression of its downstream targets. For this reason, our findings could provide additional evidence that FMRP may modulate multiple regulations of gene expression during corticogenesis.

In summary, we discovered that our FMRP “core” target genes were involved in Ras/MAPK, Wnt/β-catenin, and mTOR signaling pathways, all of which are pivotal in brain development. Proper regulation of these genes by FMRP is thus believed to be essential for appropriate corticogenesis. There could be other modulations due to the loss of function of FMRP at the epigenetic level. Our study sheds light on the significance of genetic programs in early brain development, in addition to previously proven roles in the function of postnatal neurons, concerning the etiology of FXS, of which symptoms are shared with ID and ASD.

## Methods

### Animals

Animal experiments were carried out in accordance with the National Institutes of Health guidelines outlined in the Guide for the Care and Use Laboratory Animals. The Committee for Animal Experimentation of Tohoku University Graduate School of Medicine (2017-MDA-189) and the Animal care and Use Committee of Yokohama City University (TA-16–006) approved all the experimental procedures. Male WT (C57BL/6 J) and *Fmr1* KO (B6.129P2-*Fmr1*^*tm1Cgr*^/J, stock #003,025, The Jackson Laboratory) [5] mice were used in this study. Hemizygote (*Fmr1*^*−/y*^) male and heterozygote (*Fmr1*^±^) female mice were mated to obtain WT (*Fmr1*^+*/y*^) and *Fmr1* KO (*Fmr1*^*−/y*^) male embryos.

### DNA extraction and *Fmr1* genotyping

Genomic DNA was extracted from the tail of E15.5 mouse embryos, and a standard polymerase chain reaction was performed as previously described [74, 75]. Screening for the presence or absence of the wild-type allele was performed using primers S1m (5′-GTGGTTAGCTAAAGTGAGGATGATAAAGGGTG-3′) and S2m (5′-CAGGTTTGTTGGGATTAACAGATCGTAGACG-3′). Primers N2c (5′-CGCCTCAGAAGCCATAGAGCC-3′) and N3 (5′-CATCGCCTTCTATCGCCTTCTTGAC-3′) were used to screen for the presence of the knockout allele. The amplified PCR products were visualized by electrophoresis on 1% agarose gels using the Gel Doc™ EZ Imager (Bio-Rad).

### Immunohistochemistry

Immunohistochemistry was performed as described previously [74, 75]. The sections were incubated with primary antibodies diluted with 3% BSA/TBST (containing 0.1% Triton X100), including goat anti-FMRP (1:1000; LS-B3953; LifeSpan Biosciences Inc.), rabbit anti-mTOR (1:1000; 7C10; Cell Signaling Technology), rabbit anti-phospho-mTOR (Ser2448) (1:1000; 2971; Cell Signaling Technology), mouse anti-Ctnnb1 (1:2000; 610153; BD Biosciences), and rabbit anti-Nf1 (1:1000; ab17963; Abcam) overnight at 4 °C. The secondary antibodies used were Cy3-conjugated donkey anti-goat IgG (1:500; Life Technologies), Cy3-conjugated donkey anti-rabbit IgG (1:500; Life Technologies), and Alexa 488-conjugated donkey anti-mouse IgG (1:500; Life Technologies), and counterstained with 4′,6-Diamidino-2-phenylindole dihydrochloride (DAPI)/TBST (1:1000; Sigma). Images were visualized by a confocal laser microscope Zeiss LSM800 (Carl Zeiss).

### In utero electroporation into the mouse embryonic brain

In utero electroporation was performed as described previously with minor modification [76, 77]. The expression vectors pCAG-EGFP plasmid (kindly gifted from Prof. Tetsuichiro Saito, Chiba University, Japan) and 1% Fast green in PBS were injected into the lateral ventricle of embryos at E13.5. The embryos were collected at E14.5 for analysis for FMRP localization.

### Preparation of RNA libraries and sequencing

Following the manufacturer’s protocol, RIP assay was performed to extract FMRP-bound mRNAs (n = 3, FMRP-IP; n = 2, negative control (IgG-IP)) from E14.5 WT mice cortex by using RiboCluster Profiler™ RIP-Assay Kit with anti-FMRP human polyclonal antibody, RN016P (Medical and Biological Laboratories Co., Ltd.) and Dynabeads™ Protein beads G/A (Invitrogen™). The quality and quantity of the total RNA were evaluated using the Agilent 2100 Bioanalyzer with RNA 6000 Pico Kit (Agilent). Total RNA concentration greater than 50 ng with an RNA Integrity Number (RIN) value greater than or equal to 7.9 was sequenced.

### Sequence alignment and estimation of gene expression levels

Raw reads were cleaned by removing adapter sequences and low-quality sequences (Phred quality score: 33; minimum threshold: 20; minimum length: 70) using the FASTX-Toolkit (http://hannonlab.cshl.edu/fastx_toolkit/). Using TopHat (http://tophat.cbcb.umd.edu/), cleaned reads were aligned to reference genome *Mus* *musculus* genome (mm10) with default parameter values, except for the distance between mate pairs (*r* = 200). Calculation of gene expression in FPKM and test of significance were calculated using Cuffdiff (http://cufflinks.cbcb.umd.edu/). FMRP mRNA targets were defined as transcripts showing significant difference at q < 0.01 (between FMRP-IP and IgG-IP), log_2_FC greater than 1, and FPKM value greater than 10 in the FMRP-IP samples.

### Gene ontology and protein association network

Functional annotation of the differentially expressed genes was performed using the VLAD tool (v1.6.0) of the MGI [25] and Network Analyst—a visual analytics platform for comprehensive gene profiling and meta-analysis [78]. GO was determined via an enrichment analysis (biological process), and false discovery rate (FDR) less than 0.05 were considered as significantly enriched GO annotation.

### Gene sets associated with neurogenesis, ID, and ASD

The 1791 neurogenesis genes were retrieved from the MGI database (retrieved on September 24, 2019) using GO: neurogenesis [25]. The 1088 ID genes were retrieved from the OMIM database (retrieved on September 24, 2019) using the keyword ID [27]. The 1025 ASD-associated genes were retrieved from the SFARI database (updated on May 19, 2020) [29].

### RNA extraction and RT-qPCR

Total RNA was isolated with the RNeasy Mini Kit (Qiagen) according to the manufacturer’s protocol and inverse transcribed into complementary DNA (cDNA) using the SuperScipt III™ First-Strand Synthesis System for RT-PCR (Invitrogen). RT-qPCR was performed using 2 × SsoAdvanced Universal SYBR®Green Supermix (Roche) and the Mastercycler® ep Gradient Realplex 2 (Eppendorf). The relative expression of each target was calculated (2^ΔΔCt) with *Rplp0* as normalizer. PCR sequences for RT-qPCR (Additional file 4: Table S4) were obtained in the Primerbank [79] and from a previous report [80].

### Immunoblotting

To assess protein levels, neocortical lysates from pooled (n = 2) dorsal telencephalon of WT and *Fmr1* KO embryos at E15.5 were prepared using cell lysis buffer containing 20 mM HEPES pH 7.5, 20% glycerol, 400 mM NaCl, 1 mM MgCl_2_, 0.5 M DTT, 0.5 mM PMSF, 0.1% NP40, 1 × protease and phosphatase inhibitor, and 1 mM EDTA pH 8.0. Following the manufacturer’s protocol, protein concentration was measured by the Lowry Assay Method (Bio-Rad). The neocortical lysates (25 μg) were subjected to SDS/PAGE (7.5% TGX™ FastCast™ Acrylamide Kit; Bio-Rad) and transferred onto polyvinylidene difluoride membranes (Millipore) with 40 V at 4 °C for 4 h. The membranes were then blocked in 10% TBS blocking buffer (Licor) for 1 h, and incubated with a primary antibody (as described above, Immunohistochemistry). The membrane was washed with TBST (containing 0.1% Tween 20) for 1 min with three repeats and 5 min with three repeats and incubated with a secondary antibody, either donkey anti-rabbit 680 (1:10,000; Licor), or donkey anti-mouse 680 (1:20,000; Licor), diluted in 10% TBS blocking buffer for 1 h at RT under a shaded condition. The signal was detected using the ODYSSEY infrared imaging system (Licor) and quantified using ImageJ 1.48v software (National Institute of Health) with Gapdh as normalizer.

### Statistical analysis

Data were compiled using Microsoft Excel 2011, and Student’s *t*-test was used to calculate statistical significance. Hypergeometric distribution was calculated using the webtool Hypergeometric Distribution Calculator (https://keisan.casio.com/exec/system/1180573201). Values of p < 0.05 were considered statistically significant.

## Supplementary Information

**Additional file 1: Table S1.** 865 FMRP target genes identified using RIP-seq.

**Additional file 2: Table S2.** Gene ontology (biological process) analysis of the 118 shared genes.

**Additional file 3: Table S3.** Hypergeometric distribution analyses between FMRP target genes.

**Additional file 4: Table S4.** Primer sequences used for RIP-qPCR and RT-qPCR.

## Data Availability

The datasets generated and/or analyzed during the current study are available in public databases (MGI, OMIM, and SFARI) and are included in this published article. Additional inquiries can be directed to the corresponding author.

## Abbreviations

ASD: Autism spectrum disorder
BSA: Bovine serum albumin
cDNA: Complementary DNA
CP: Cortical plate
DAPI: 4′,6-Diamidino-2-phenylindole dihydrochloride
DTT: Dithiothreitol
FDR: False discovery rate
FMRP: Fragile X mental retardation protein
*Fmr1*: *Fragile* *X* *mental* *retardation* *1*
*Fmr1*KO: *Fmr1* Knockout
FPKM: Fragment per kilobase of transcript per RNA-seq read mapped
FXS: Fragile X syndrome
GO: Gene ontology
HEPES: (2-Hydroxyethyl)-1-piperazineethanesulfonic acid
ID: Intellectual disability
Log_2_FC: Fold change on a logarithmic scale with base twofold-change
MGI: Mouse Genome Initiative
NPCs: Neural stem/precursor cells
OMIM: Online Mendelian Inheritance in Man
PCR: Polymerase chain reaction
PMSF: Phenylmethylsulfonyl fluoride
RBP: RNA-binding protein
RGCs: Radial glial cells
RT-qPCR: Real-time quantitative PCR
RIP: RNA immunoprecipitation
RIP-seq: RNA immunoprecipitation-sequencing
SDS-PAGE: Sodium dodecyl sulfate polyacrylamide gel electrophoresis
SFARI: Simons Foundation Autism Research Initiative
TBST: Tris-buffered saline, 0.1% Tween 20 for Western blot; Tris-buffered saline, 0.1% Triton X for immunohistochemistry
VZ: Ventricular zone
WT: Wild type
